# Histological evaluation of capsules formed by texturized silicone implants with and without polyester mesh coverage (Parietex®). A study on female rats

**DOI:** 10.1590/ACB360505

**Published:** 2021-06-14

**Authors:** Ralf Berger, Jurandir Marcondes Ribas, Osvaldo Malafaia, Paulo Afonso Nunes Nassif, Eduardo Nascimento Silva, Alfredo Benjamin Duarte da Silva, Milka Takejima, Marcelo Augusto de Souza, Pedro Henrique de Paula, Mário Rodrigues Montemor, Lucia de Noronha

**Affiliations:** 1 Fellow Master degree. Postgraduate Program in Principles of Surgery - Mackenzie Evangelical School of Medicine – Curitiba (PR), Brazil.; 2 PhD, Associate Professor. Postgraduate Program in Principles of Surgery - Mackenzie Evangelical School of Medicine – Curitiba (PR), Brazil.; 3 PhD, Full Professor. Postgraduate Program in Principles of Surgery - Mackenzie Evangelical School of Medicine – Curitiba (PR), Brazil.; 4 PhD. General Surgery – Universidade Estadual de Ponta Grossa – Ponta Grossa (PR), Brazil.; 5 MD. Plastic Surgery Department - Hospital Erasto Gaertner – Curitiba (PR), Brazil.; 6 MD. Postgraduate Program in Plastic Surgery - Hospital Universitário Evangélico Mackenzie, and Instituto de Pesquisa Médica – Curitiba (PR), Brazil.; 7 Graduate Student. Universidade Estadual de Ponta Grossa - Ponta Grossa (PR), Brazil.; 8 MSc. Clinical Surgery – Universidade Federal do Paraná – Curitiba (PR). Assistant Professor. Anatomical Pathology – Universidade Estadual de Ponta Grossa - Ponta Grossa (PR). Head. Department of Anatomical Pathology - Santa Casa de Misericórdia - Ponta Grossa (PR), Brazil.; Universidade Estadual de Ponta Grossa, Ponta Grossa, PR, Brazil; Santa Casa de Misericórdia, Department of Anatomical Pathology, Ponta Grossa, PR, Brazil; 9 PhD. School of Medicine – Pontifícia Universidade Católica do Paraná – Curitiba (PR), Brazil.

**Keywords:** Breast Implants, Prostheses and Implants, Mammaplasty, Rats

## Abstract

**Purpose:**

To evaluate capsules formed by microtextured silicone implants with and without Parietex® mesh coverage histologically.

**Methods:**

Sixty Wistar rats were divided in two groups (meshed and unmeshed). Each group was, then, divided into two subgroups for evaluation at 30 and 90 days. Capsules were analyzed based on hematoxylin and eosin (HE) and picrosirius staining.

**Results:**

The number of fibroblasts, neutrophils and macrophages was similar among all subgroups. There was a higher lymphocyte reaction in the 30-day meshed group (p = 0.003). Giant cell reaction, granulation tissue and neoangiogenesis were similar among the subgroups. Synovial metaplasia was milder at 90-day in the unmeshed (p = 0.002) and meshed group (p < 0.001). Capsular thickness was significantly greater in the meshed samples (30-day p < 0.001 and 90-day p < 0.001). There was a similar amount of collagen types I and III in both groups.

**Conclusions:**

The mesh-covered implants produced capsules similar to the microtextured ones when analyzing inflammatory variables. Synovial metaplasia was milder at 90 than at 30 days, and the capsular thickness was significantly greater in the meshed group. A similar amount of collagen types I and III was observed. Due to these characteristics, the mesh coverage did not seem to significantly affect the local inflammatory activity.

## Introduction

Breast reconstruction can be performed with autologous techniques, using the patient’s own tissues, which is generally cited as the standard procedure^1^. Autologous reconstruction, however, may not be possible in some patients. For example, thin women may not have enough abdominal tissue to enable the reconstruction with rectus abdominis muscle flap^2^. In addition, some women might not be willing to accept the donor site morbidity, extended operative and recovery time, inherent to autologous reconstruction. The presence of comorbidities can also limit the options for reconstruction^3^.

The alternative to autologous reconstruction is the implant-based surgery, in which an important restriction is the inadequate soft tissue coverage, which can lead to skin damage, implant exposure, poor aesthetic results and asymmetry^4^.

An alternative to provide tissue coverage is the acellular dermal matrix, which provides an extra layer and support for the lower pole of the reconstructed breast. The acellular dermal matrix has also reduced complications such as visibility of implant ripples, unstable position^5^ and capsular contracture^6^.

Although well established in the literature, the use of acellular dermal matrix is expensive, often prohibitive in Brazil. Therefore, the use of synthetic meshes may be a low-cost option.

The use of meshes made by different materials has been increasingly applied during immediate breast reconstruction with silicone implants. The complication rates when using polypropylene and titanium meshes on silicone implants seem to be similar to those observed in pure silicone implants. However, the use of synthetic meshes entails new scenarios and the demand for surgeons to recognize new complications and their histological behavior^7^, since there is lack of knowledge regarding inflammatory alterations on meshes associated with silicone implants.

According to some authors, a capsular contracture with clinical symptoms is related to local inflammatory activity^8^-^10^. Several studies have successfully evaluated the use of meshes during breast reconstruction with implants^11^-^24^. However, the histological behavior of Parietex Composite® (Covidien, Boulder, United States) associated with silicone implants is not known.

The aim of this study was to evaluate the capsules formed around silicone implants with and without a Parietex Composite® coverage histologically, assessing the mesh effect on inflammatory variables, synovial metaplasia, capsular thickness and collagen types I and III.

## Methods

This study was carried out in the *vivarium* and in the Laboratory of Operative Technique and Experimental Surgery at Universidade Estadual de Ponta Grossa (protocol numbers 13,252/2018 and 3,973/2018), after being approved by the Ethics Committee on the Use of Animals (CEUA), process number 032/2018.

This is a primary interventional prospective non-randomized study. No calculations were performed for the sample size, obtaining a smaller sample based on already published studies similar to this one, facilitating the process of acceptance by the CEUA.

Sixty albino rats (*Rattus norvegicus*) weighing between 200 and 300 grams, 100 days old, of Wistar strain, were used. The 60 animals were distributed in two groups of 30 rats each (implants with and without mesh coverage), and each group was divided into two subgroups, to be evaluated at 30 and 90 days. Four rats were allocated per 450-cm^3^ acrylic box, lined with wood shavings. They had free access to water and a specific diet for the species, *ad libitum*, in addition to alternating light in 12-hour cycles at room temperature.

By the date of the first euthanasia, with 30 days, eight animals in the unmeshed group and five in the meshed group died. One animal from each group was excluded due to the lack of quality of the piece, and two animals from the meshed group by rotation of the mesh-implant set. After that, the following distribution was made (Table 1):

**Table 1:** Final distribution of animals in groups and subgroups.

Groups	Subgroups	
	30 days	90 days
Unmeshed	10 animals	11 animals
Meshed	10 animals	12 animals

### Implanted materials

LifeSil® (Curitiba, PR, Brazil) implants were used, which have the same characteristics as micro-texture implants, except that they are not filled with silicone, constituted only by the 20-mm-diameter microtextured implant cover.

The Parietex Composite® mesh, used to cover the outer surface of the implants in one of the groups, consisted of three-dimensional multifilament polyester with an absorbable, continuous and hydrophilic film on one side. The film consists of porcine collagen, polyethylene glycol and glycerol.

### Surgical procedure

The animals were anesthetized with intraperitoneal injection of ketamine 10%, 80 mg/kg, and xylazine 2%, 10 mg/kg. No fasting was performed, and they were placed in prone position after trichotomy.

A 1,5 -cm-long incision was made in the posteroinferior costal margin, in the midline. The implant pocket was round, with a 5-mm margin from the implants.

The implants were positioned 5 mm from the incision. On the meshed implants, the matrix was positioned on the dorsal side. The suture was performed with four stitches, Prolene® 5.0 (Ethicon, Somerville, New Jersey, United States), and there were no dressings.

Postoperative analgesia was performed with two subcutaneous doses of ketoprofen 5 mg/kg, with an administration interval of 24 hours.

Euthanasia was performed with triple the therapeutic dose of Cetamin®/240–270 mg/kg and Xilazin®/30–40 mg/kg intraperitoneally, followed by cervical dislocation.

### Histological evaluation

#### Hematoxylin and eosin staining

The procedure was used for the evaluation of inflammatory variables, synovial metaplasia, and capsular thickness.

#### Picrosirius coloring

This technique was used to assess the amount of collagen types I and III. The software AxioVision® 4.9.1.0 (Zeiss, Oberkochen, Germany) was used to obtain the images. The percentage of collagen types I and III was measured using semi-automatic segmentation, in the Image Proplus® 4.5 morphometry program (Media Cybernetics, Rockville, MD, United States).

**Table 2:** Percentage of cases with moderate/intense classification according to the group (meshed or unmeshed) and subgroup (30 days or 90 days).

Variable	Subgroup	Group		p* (unmeshed × meshed)
		Unmeshed	Meshed	
Fibroblasts	30 days	20%	70%	0.070
	90 days	27.3%	16.7%	0.640
	p* (30d × **90d)**	1	0.030	
Neutrophils	30 days	20%	0%	0.474
	90 days	0%	0%	1
	p* (30d × **90d)**	0.213	1	
Macrophages	30 days	0%	0%	-
	90 days	0%	0%	-
	p* (30d × **90d)**	-	-	
Lymphocytes	30 days	30%	100%	0.003
	90 days	45.4%	91.7%	0.027
	p* (30d × **90d)**	0.659	1	
Granulation tissue	30 days	10%	0%	1
	90 days	0%	0%	1
	p* (30d × **90d)**	0.472	1	
Neoangiogenesis	30 days	10%	10%	1
	90 days	0%	16.7%	0.478
	p* (30d × **90d)**	0.476	1	
Synovial metaplasia	30 days	80%	90%	1
	90 days	9.1%	8.3%	1
	p* (30d × **90d)**	0.002	<0.001	

#### Statistical analysis

For each of the variables, the groups with and without mesh coverage were compared, in the 30 and 90-day subgroups. Then, the subgroups were compared with one another.

The results were described by averages, standard deviations, medians, minimum and maximum values (quantitative variables) or by frequencies and percentages (categorical variables). Fisher’s exact test was used for inflammatory variables, the Mann-Whitney non-parametric test for capsular thickness and Student’s t test for comparison in relation to the percentage of collagen. The significance level of 0.05 was adjusted by applying the Bonferroni correction (p < 0.012). The data were analyzed with the Stata/SE® v. 14.1 (Stata Corporation LLC, College Station, TX, United States) software.

## Results

### Hematoxylin and eosin staining

Only the variables with statistical significance were highlighted in the pictures. Table 2 shows the percentage of rats that had each characteristic evaluated as moderate or accentuated.

Giant cell reaction was analyzed only as present or absent. All animals of all groups had the presence of this variable.

### Fibroblasts

In the unmeshed group, in both subgroups (30 and 90 days), most animals had a mild presence. In the meshed group, the majority had a moderate presence at 30 days and a mild presence at 90 days. Although the 30 and 90-day unmeshed subgroups and the 90-day meshed subgroup showed a mild presence, no statistical significance was obtained.

### Neutrophils

The majority of the animals, in both groups, had a mild presence. No significant differences were found between the two groups in the different subgroups.

### Macrophages

This variable had a mild presence in both groups, in all analyzed animals. Thus, there was no statistical comparison.

#### Lymphocytes

In the unmeshed group, the presence was mild in the 30-day subgroup, whereas in the meshed group the majority of the animals exhibited a moderate or intense presence of this variable in both subgroups.

**Figure 1:**
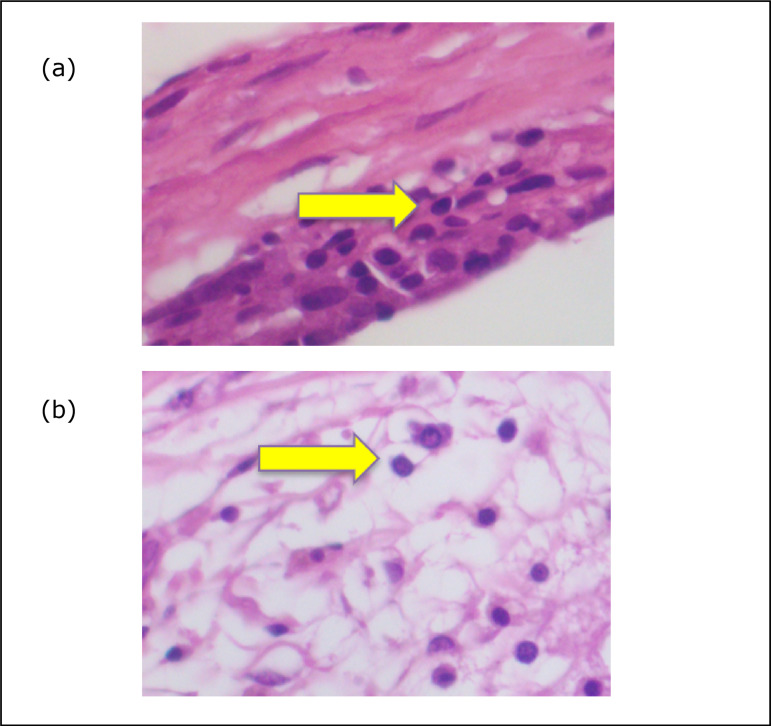
Photomicrography of microtextured implant (**a**) and meshed implant (**b**), showing lymphocytes.

When comparing the 30-day meshed and unmeshed groups, statistical significance was obtained (p = 0.003) (Fig. 1).

### Giant cell reaction

This reaction was only analyzed as absent or present, and all animals in the four subgroups had this characteristic. Thus, there was no statistical comparison.

### Granulation tissue

The vast majority of animals had a mild presence of this variable. When the groups and subgroups were compared, there was no statistical significance.

### Neoangiogenesis

In all subgroups, the majority of the rats had a mild presence of the variable. When the groups and subgroups were compared with one another, there was no statistical significance.

### Synovial metaplasia

**Figure 2:**
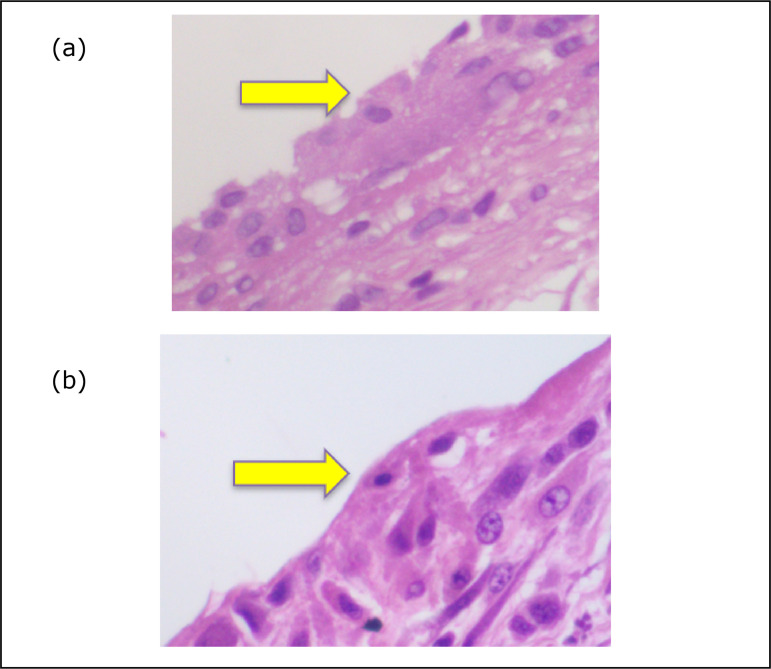
Photomicrography of microtextured implant(**a**) and meshed implant (**b**), showing synovial metaplasia.

In the 30-day subgroups, a moderate or intense presence of this variable was found in most animals, while in the 90-day subgroups most of them had a mild presence. In both groups, when comparing 30 and 90-day subgroups, there was statistically significant difference (unmeshed p = 0.002/meshed p<0.001) (Fig. 2).

### Capsule thickness

**Figure 3:**
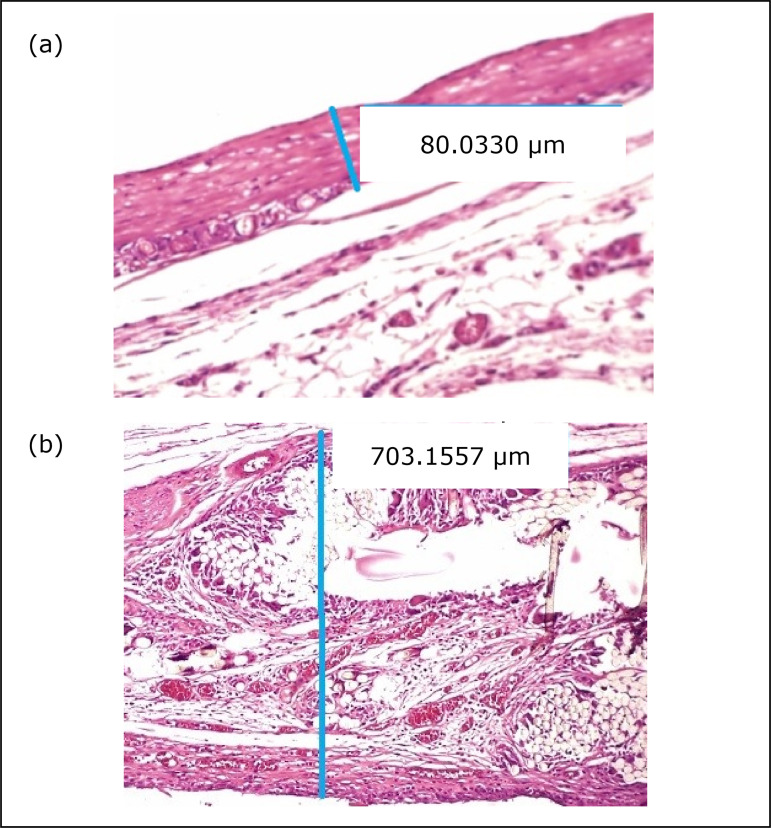
Photomicrography of microtextured implant **(a)** and meshed implant **(b)**, showing capsular thickness (magnification x20).

This finding was lower in the unmeshed compared to the meshed group, with statistical significance (30 days p < 0.001 / 90 days p < 0.001) (Fig. 3).

Table 3 contains the median with the minimum and maximum values of capsular thickness. Table 4 contains p-values.

**Table 3:** Median, minimum and maximum values of the capsule thickness (μm) according to the group (meshed or unmeshed) and the subgroup (30 and 90 days).

Variable	Subgroup	Group	
		Unmeshed	Meshed
Thickness	30 days	70.4 (35.3–144)	683.3 (566.7–766.7)
	90 days	56.7 (32.3–93.7)	633.3 (500–700)

**Table 4:** Compared groups and subgroups in relation to capsular thickness with p-value.

**30 days**	unmeshed × meshed			<0.001
**90 days**	unmeshed × meshed			<0.001
**Unmeshed**	30 days × 90 days			0.387
**Meshed**	30 days × 90 days			0.030

Mann-Whitney non-parametric test, p < 0.012 (Bonferroni correction).

### Picrosirius staining

#### Collagen types I and III

**Figure 4:**
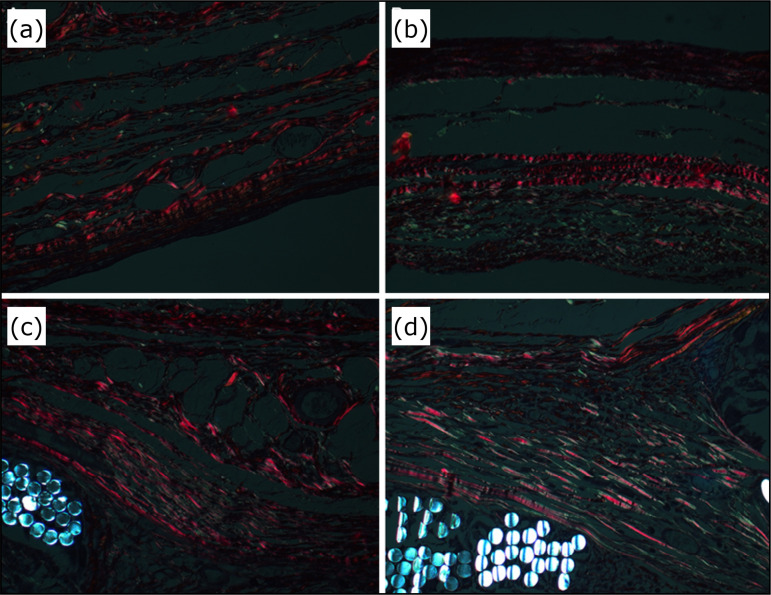
Photomicrography evidencing collagen fibers. **(a)** 30-day unmeshed group, **(b)** 90-day unmeshedgroup, **(c)** 30-day meshed group, **(d)** 90-day meshed group.

Note: *Red:* type I collagen; *Green:* type III collagen; *Blue:* mesh (picrosirius staining, magnification x400, polarized light).

The Fig. 4 shows type I collagen in reddish color and type III collagen in greenish color. In the meshed group, the matrix is exhibited by the bluish color.

In the unmeshed group, in both subgroups, the averages were slightly higher for collagen type I. However, when the groups and subgroups were compared with one another, no statistical significance was found (Tables 5 and 6).

**Table 5:** Descriptive statistics of collagen type I according to the subgroups.

Group	Days	n	Collagen I (%)				
			Avg.	Median	Min.	Max.	SD
Unmeshed	30	10	63.2	71.6	24.4	82	20.7
Meshed		10	53.5	51	40.2	75.9	10.9
Unmeshed	90	11	64.6	68	46.8	88	12.2
Meshed		12	50	49.9	26.2	69	13.7

Avg: Average; Min: Minimum; Max: Maximum; SD: Standard Deviation

**Table 6:** Compared groups and subgroups in relation to collagen type I with p-value.

**30 days**	unmeshed × meshed			0.209
**90 days**	unmeshed × meshed			0.012
**Unmeshed**	30 days × 90 days			0.843
**Meshed**	30 days × 90 days			0.519

Student’s t test for independent samples, p < 0.012 (Bonferroni correction).

**Figure 5:**
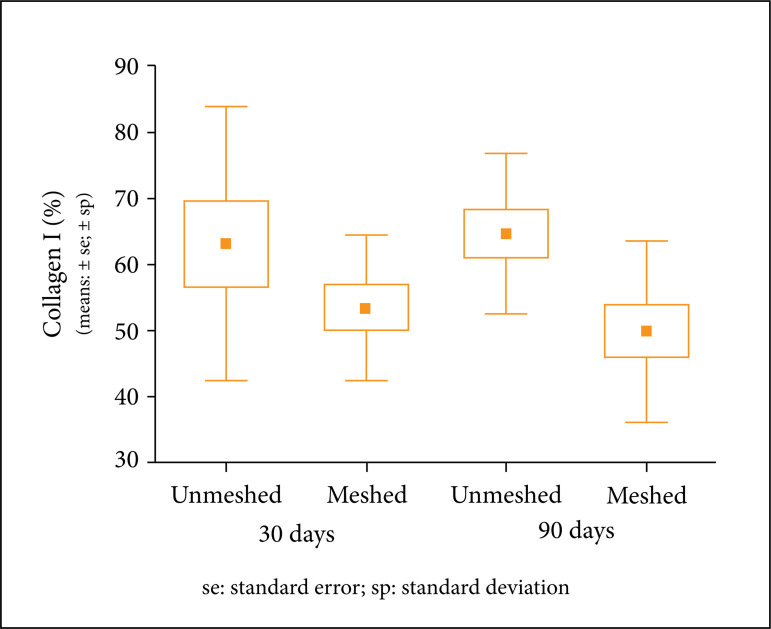
Average, standard errors and standard deviations of area percentages with collagen type I in each subgroup.

The following graphic shows that collagen type I was similar between groups (Fig. 5).

The amount of collagen type III was similar between groups and subgroups. When the groups and subgroups were compared, there was no statistical significance (Tables 7 and 8).

**Table 7:** Descriptive statistics of collagen type III according to the subgroups.

Group	Days	n	Collagen III (%)				
			Avg.	Median	Min.	Max.	SD
Unmeshed	30	10	36.8	28.4	18	75.6	20.7
Meshed		10	46.5	49	24.1	59.8	10.9
Unmeshed	90	11	35.4	32	12	53.2	12.2
Meshed		12	50	50.1	31	73.8	13.7

Avg: Average; Min: Minimum; Max: Maximum; SD: Standard Deviation

**Table 8:** Compared groups and subgroups in relation to collagen type III with p-value.

**30 days**	unmeshed × meshed			0.209
**90 days**	unmeshed × meshed			0.012
**Unmeshed**	30 days × 90 days			0.843
**Meshed**	30 days × 90 days			0.519

Student’s t test for independent samples, p<0.012 (Bonferroni correction).

**Figure 6:**
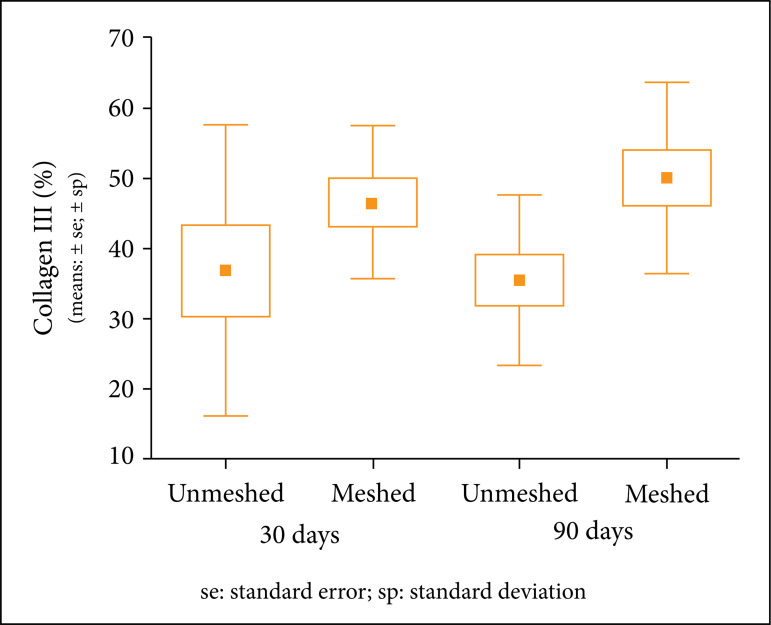
Average, standard errors and standard deviations of area percentages with type III collagen in each subgroup.

The Fig. 6 shows that type III collagen was similar between the groups.

## Discussion

The rat (*Rattus norvegicus albinus*) chosen by the authors is the most used animal in capsular contracture studies, for presenting easy reproducibility of results and resistance to surgical procedures^25^-^27^.

Due to the difficulty in obtaining large animal samples for research in our institution, we have based our sample size on already published studies similar to this, which also used animals for experimentation. Thus, no calculations were performed for the sample size, obtaining a smaller sample, facilitating the process of acceptance by the CEUA. Since it’s a small sample, there may have been a loss of statistical power in the analysis of some variables. Despite differing percentages in their values, we need to rely on the stipulated significance range (p<0.012 with Bonferroni correction) to complete the analysis, even though there are increased chances of false negative results.

Following Mendes *et al*.^26^, subcutaneous dissection was carried out, superficial to the *panniculus carnosus*, on the rats’ back, unlike Silva *et al*.^28^ and Silva *et al.* 29, who performed deeper dissection at this plane.

The coverage of silicone implants in breast reconstruction is usually carried out with acellular dermal matrix^6^,^18^. However, the use of meshes might be as safe as and presents lower cost.

The reason why the authors chose to use a synthetic mesh in this study is that several papers already reported successful use of this material when associated to silicone implants in human beings^11^-^24^.

In a recent study with Parietex® mesh, the same chosen for this study, Parietex® mesh was compared to other types of synthetic meshes and presented less intense fibrosis than the ones of polypropylene mesh^30^.

Capsular thickness and the progression to a contracture with clinical symptoms in breast implants are proportional to the inflammatory activity^8-0,31^. Bui *et al*.^32^ investigated the relation between the capsule histology and the contracture clinical symptoms and concluded that the contracture development is related to an increase in the capsular thickness, the alignment of collagenous fibers, the presence of contractile microfibroblasts, and greater alpha-SMA expression.

Due to this association between inflammation, capsular thickness and contracture, we opted for analyzing the inflammatory variables when studying the Parietex® mesh.

In this study, the capsular thickness was smaller in the unmeshed group. Other authors also found smaller capsular thickness in textured implants when compared to implants that used other types of coverage, namely: Balderrama *et al*.^33^, investigating polyurethane foam; and Vieira *et al*.^34^ and Silva *et al*.^28^, evaluating polyurethane coating.

This research is in disagreement with Bergmann *et al*.^25^, who found smaller capsular thickness in implants covered with titanium mesh, TiLOOP^®^ (PFM Medical, Köln, Germany), when comparing them with textured implants.

The presence of fibroblasts in the capsules ranged from discrete to moderate, which is in agreement with Haddad Filho *et al*.^35^, who compared textured implants with PTFE-E-covered implants. This study disagrees with Bergmann *et al*.^25^, who reported greater number of myofibroblasts in capsules of TiLOOP^®^-covered implants when comparing them with textured implants in 60-day subgroups.

Haddad Filho *et al*.^35^ found a higher number of neutrophils in the mesh-covered group at 30 days, unlike this study, that did not find differences in the neutrophil count.

All capsules under analysis presented similar number of macrophages, which is in agreement with other studies^33^,^35^.

In the meshed group, the number of lymphocytes was higher at 30 days, contradicting Haddad Filho *et al*.^35^, who found similar numbers between the textures and PTFE-E covered groups.

Giant cell reaction was observed in all samples, which is in agreement with other studies that compared textured implants to implants using different types of coverage^26^,^27^,^33^,^35^. Unlike Silva *et al*.^28^, who found a moderate to intense presence of giant cell reaction in polyurethane-covered samples, it seems relevant to emphasize that in this study this variable was only classified as present or absent.

The granulation tissue was present in a discrete way, without differences between groups, in accordance with other studies^9^,^33^,^35^. This result is in disagreement with Silva *et al.* 28, who found intense formation of granulation tissue in polyurethane implants.

Neovascular formation was essentially mild in all subgroups, corroborating findings by Silva *et al*.^28^ in subgroups from the same evaluation period. This result opposes to the one by Haddad Filho *et al*.^35^, who found, in the unmeshed subgroup, greater intensity of vascular formation in the 90-day subgroups when compared to the 30-day subgroups. Those authors also reported higher neoangiogenesis in the PTFE-E group at 30 days. Other authors also found more intense neovascularization in the presence of another coverage in addition to the textured one^27^,^34^.

Bergmann *et al*.^25^, however, reported intense neovascular formation in the textured group when compared to the titanium-covered mesh group.

This study partially agrees with Prantl *et al*.^9^, who evaluated implant capsules in humans and found the presence of synovial metaplasia in most of them, whereas in this study this variable was present in all animals of both groups.

Unlike Basseto *et al*.^36^, who found similar synovial metaplasia between the subgroups, this study showed a more pronounced presence of this characteristic in the 30-day subgroups.

The moderate and accentuated presence of synovial metaplasia at 30 days and mild at 90 days differs from the findings by Silva *et al*.^28^, who detected an absent or mild presence throughout the evaluation period, despite the fact that those researchers compared textured implants with polyurethane implants.

The findings of this study are close to those found by Hansson *et al*.^24^, who compared the use of biological and synthetic meshes and reported the presence of synovial metaplasia in most cases, since in this research this variable was observed in all cases.

Alterations in collagenous fibers might be present in capsular contracture cases^31^,^32^,^37^. Therefore, they were analyzed in this study. Brazin *et al*.^38^ studied patients with grade IV Baker contracture and concluded that the capsule collagenous production by fibroblasts is mediated by mastocytes.

In agreement with Minami et al., who observed a slight increase in collagen type III in the textured implant group, in this study the results in the unmeshed group (microtextured implant) in the 30 and 90-day subgroups were similar. Those authors also found a slight decrease in collagen type III in the textured group from 30 to 90 days. Similar results were found in this study in the 30 and 90-day subgroups.

This study disagrees with Balderrama *et al*.^33^, who found a significant decrease in the amount of type III collagen in the textured group in the 30 to 60-day subgroups, because in this study type III collagen remained similar in the unmeshed sample. Those authors also found a significant increase in the amount of type I collagen in the subgroup from 30 to 60 days, whereas in this study, in the unmeshed group, a similar amount of type I collagen was found in the subgroups analyzed.

Differing from Silva *et al*.^28^, the percentage of collagen types I and III was similar between textured implants and those with additional coating in all subgroups analyzed, despite the fact that those researchers used polyurethane implants for comparison. Due to these characteristics, themesh coverage did not seem to significantly affectthe local inflammatory activity.

## Conclusions

The implants covered by Parietex Composite® mesh produced capsules similar to those ones found in textured implants when analyzing inflammatory variables. Synovial metaplasia was milder at 90 than at 30 days, and the capsular thickness was significantly greater with the mesh coating. A similar amount of collagen types I and III was formed in the meshed and unmeshed implant capsules. Due to these characteristics, the mesh coating did not seem to significantly affect the local inflammatory activity.
